# Chlorogenic Acid Alleviates Heat Stress-Induced Fetal Growth Restriction Through Gut–Placental Crosstalk by Reshaping Gut Microbiota and Mediating Keap1-Nrf2 Antioxidant Signaling

**DOI:** 10.3390/antiox15081036

**Published:** 2026-08-20

**Authors:** Chenjun Wang, Chang Yan, Siyu Wu, Biyan He, Yongtian Yin, Caixue Xu, Yiling Xiang, Yifei Zhang, Jiangong Li, Yingjie Wu, Ning Liu, Yinghe Qin

**Affiliations:** 1 State Key Laboratory of Animal Nutrition and Feeding, China Agricultural University, Beijing 100193, China; wangchenjun@cau.edu.cn (C.W.); yanchang@cau.edu.cn (C.Y.); s20253040936@cau.edu.cn (S.W.); s20243040882@cau.edu.cn (B.H.); a05220200@neau.edu.cn (Y.Y.); 20220200217@stu.qau.edu.cn (C.X.); zxy13098561165@email.swu.edu.cn (Y.X.); zhangyifei@cau.edu.cn (Y.Z.); jli153@cau.edu.cn (J.L.); wuyingjie@cau.edu.cn (Y.W.); 2 Beijing Advanced Innovation Center for Food Nutrition and Human Health, China Agricultural University, Beijing 100193, China

**Keywords:** fetal growth restriction, chlorogenic acid, Keap1-Nrf2, gut–placenta axis

## Abstract

Background: Climate change-driven heat stress (HS) poses a growing threat to pregnancy outcomes; however, the mechanisms linking maternal HS to fetal growth restriction (FGR) remain incompletely understood, and effective nutritional interventions are lacking. The gut–placenta axis critically governs maternal–fetal health, but its role in mediating HS-related FGR has yet to be elucidated. Methods: A murine model of gestational HS (38.5 °C, 2.5 h daily from E0.5 to E12.5) was established to investigate the protective effects of chlorogenic acid (CGA) on HS-induced FGR. Placental efficiency, oxidative status, barrier integrity, and intestinal permeability were assessed. Gut microbiota composition was profiled by 16S rRNA sequencing, while tight junction protein expression in the placenta and intestine was evaluated by Western blot. Additionally, antibiotic-induced microbiota reduction and fecal microbiota transplantation (FMT) were performed to explore the role of gut microbiota in alleviating HS. Results: HS exposure significantly reduced fetal weight without altering litter size, accompanied by reduced placental efficiency and proportion of the labyrinth zone relative to the total placental area. HS triggered oxidative stress and downregulated tight junction proteins (Claudin-1, Occludin) in both the placenta and intestine, indicating compromised barrier integrity. CGA treatment robustly reversed these abnormalities and restored placental structure. In the gut, CGA prevented HS-induced intestinal barrier dysfunction and selectively reshaped the microbiota, enhancing the Firmicutes/Bacteroidetes ratio and promoting beneficial Lactobacillus lineages. Notably, under antibiotic-induced microbial reduction, FMT from CGA-treated donors effectively alleviated HS-induced fetal growth restriction. Conclusions: In summary, our study suggests that gestational HS exposure may trigger FGR through the gut–placenta axis. CGA effectively reverses FGR by restoring placental efficiency and placental barrier integrity, while alleviating maternal gut dysbiosis and intestinal barrier impairment. FMT experiments further confirm that the gut microbiota may play a key role in this protection. Collectively, these findings suggest that targeting the gut–placenta axis with CGA may offer a practical nutritional strategy to mitigate HS-triggered adverse pregnancy outcomes.

## 1. Introduction

Anthropogenic activities including open burning, fossil fuel combustion, deforestation, and intensive agriculture have driven significant increases in greenhouse gas emissions, accelerating global warming and climate change [1]. The concomitant rise in global temperatures has precipitated widespread heat stress (HS) across various species, severely compromising the health and productivity of plants, animals, and humans [2]. Pregnant individuals are particularly vulnerable to HS exposure, bearing a disproportionate risk of adverse gestational outcomes such as preterm birth, stillbirth, placental dysfunction, and fetal developmental anomalies [3,4,5]. Despite these severe consequences, our understanding of the underlying pathogenic mechanisms remains limited, hindering the development of efficacious intervention strategies.

To date, pioneering research has primarily focused on the impact of HS on specific physiological parameters, such as thermoregulation, oocyte competence, placental blood flow, and intestinal integrity [6,7,8]. Nevertheless, the maternal gut microbiota, acting as a pivotal systemic regulator that orchestrates the physiological status of distal organs through intestinal microenvironment homeostasis, remains vastly understudied in gestational HS research. The gut–placenta axis is paramount for optimal fetal development, mediating the transfer of gut-derived nutrients to the placenta to satisfy fetal metabolic demands [9]. Under physiological gestational conditions, the maternal gut microbiota serves as a core modulator at the host–environment interface to sustain homeostasis of nutrient metabolism and immune homeostasis [10]. Interestingly, accumulating evidence links gut dysbiosis to pregnancy-associated pathologies. For example, dysbiosis-driven bacterial translocation has been implicated in the pathogenesis of preeclampsia [11] and is associated with fetal growth restriction, suggesting a microbial etiology for diseases of placental origin [12]. Crucially, HS can compromise intestinal barrier integrity, facilitating the translocation of microbial lipopolysaccharides (LPS) into the systemic circulation and instigating low-grade systemic inflammation [13,14]. This intestinal inflammation can propagate along the gut–placenta axis, triggering a placental inflammatory response and disrupting placental barrier function, a pathological cascade implicated in 40–70% of premature births and fetal developmental anomalies [15]. Consequently, restoration of gut microbiota homeostasis and suppression of the inflammatory cascade across the gut–placenta axis constitute promising therapeutic strategies to ameliorate adverse pregnancy outcomes triggered by gestational HS.

Furthermore, gut dysbiosis is known to activate oxidative stress pathways, precipitating tissue damage in both the intestine [16] and the placenta [17]. Oxidative stress, characterized by a fundamental imbalance between reactive oxygen species (ROS) production and endogenous antioxidant defense capacity, serves as a primary driver of cellular injury during HS [18,19]. Elevated ROS levels inflict oxidative damage upon cellular macromolecules, further compromising intestinal barrier integrity and impairing placental structure and function, which ultimately culminates in adverse pregnancy outcomes [20]. Therefore, mitigating oxidative stress is a critical therapeutic imperative for preserving maternal and fetal health during maternal heat exposure. In this context, chlorogenic acid (CGA), a natural polyphenolic compound abundant in coffee, fruits, and vegetables, has emerged as a compelling therapeutic candidate due to its robust antioxidant and microbiota-modulating properties. Operating both as a direct free radical scavenger and a potentiator of endogenous antioxidant systems, CGA effectively mitigates oxidative damage in maternal and fetal tissues during pregnancy [21,22,23]. Increasingly, the biological efficacy of CGA is attributed to its capacity to modulate the gut microbiome. CGA remodels the microbial community architecture, selectively promoting the proliferation of beneficial genera (e.g., *Lactobacillus* and *Bifidobacterium*) while suppressing pathogenic taxa [24,25]. Additionally, CGA fortifies intestinal barrier integrity and modulates local mucosal immunity, notably by enhancing secretory immunoglobulin A (sIgA) production [26]. Despite these favorable biological properties, whether CGA could alleviate HS-elicited gestational complications via targeted regulation of the gut–placenta axis has yet to be investigated.

In the present study, a murine model of gestational HS was used to investigate the ameliorating effect of CGA on adverse pregnancy outcomes. We hypothesized that CGA protects against HS-induced FGR by reinforcing the maternal intestinal barrier and reshaping the gut microbiota, thereby attenuating systemic and placental oxidative stress via the gut–placenta axis. Antibiotic-induced microbial reduction and fecal microbiota transplantation (FMT) were employed to dissect the causal role of the microbiome in this process.

## 2. Materials and Methods

### 2.1. Ethics Statement

All experiments involving animals were conducted according to the ethical policies and procedures approved by the China Agricultural University Animal Care and Animal Use Committee (AW03406202-1-05).

### 2.2. Animals

The 7-week-old ICR male and female mice were purchased from Spearhead Biotech Co., Ltd. (Beijing, China). The 7-week-old mice were housed in cages and maintained under standard environmental conditions of temperature (24 ± 2 °C) and relative humidity (60%) and underwent a 1-week adaptation stage before mating. E0.5 (Embryonic 0.5 day) was defined as the morning when a vaginal plug was observed after overnight mating.

### 2.3. Heat Stress Mouse Model and CGA Administration Experiment

The pregnant mice were divided into control (CON, *n* = 6), HS (*n* = 6), and CGA (HS + 0.02%CGA *n* = 6) groups. CGA was dissolved in the drinking water at a concentration of 0.02% (*w/v*), and the solution was freshly prepared and replaced daily to ensure stability and bioactivity. Mice in the CON group were maintained under 24 ± 2 °C. Mice in the HS and CGA groups were exposed to 38.5 °C from 12:00 to 14:30 using an artificial climate chest and returned to 24 ± 2 °C for the remainder of the day. At E12.5, mice were euthanized, and intestinal, placental, and fetal tissues were harvested for subsequent analyses.

### 2.4. Hematoxylin and Eosin Staining and Placental Zonation

Paraffin-embedded mouse placentas and intestines were serially sectioned at a thickness of 6 μm. To evaluate placental and intestinal layer architecture, sections were subjected to hematoxylin and eosin (H&E) staining using a commercial kit (C0105, Beyotime, Beijing, China). Briefly, sections were deparaffinized in xylene (2 × 10 min) and rehydrated through a graded ethanol series (100%, 90%, 70%, and 50% for 5 min each). Tissues were stained with hematoxylin for 5 min, rinsed under running tap water for 15–20 min, and counterstained with eosin for 30 s. Subsequently, sections were dehydrated in an ascending ethanol series (70%, 80%, 90%, and 100% for 10 s each), cleared in xylene (2 × 3 min), and mounted with a resinous medium. The total placental area and the labyrinth zone (LZ) area were manually delineated by investigators who were blinded to the experimental groups.

### 2.5. 16S Ribosomal RNA (rRNA) Gene Sequencing and Analysis

Following euthanasia, the colon was isolated in a sterile saline solution using aseptic surgical instruments. Under a sterile surgical hood, each cecum was incised with a sterile scalpel, and the luminal contents were collected, transferred into sterile cryotubes, and immediately snap-frozen at −80 °C for subsequent analysis. The V3–V4 hypervariable region of the 16S rRNA gene was amplified using the primer pair 341F (5′-CCTACGGGNGGCWGCAG-3′) and 806R (5′-GGACTACHVGGGTWTCTAAT-3′). The resulting amplicons were then subjected to sequencing. Raw paired-end reads were processed using Cutadapt for adapter trimming and subsequently merged via FLASH. The QIIME 2 pipeline was employed for downstream quality control, denoising, and chimera depletion, yielding an amplicon sequence variant (ASV) feature table. Taxonomic annotation was performed by mapping the ASVs against the Greengenes2 database [27]. Diversity indices were calculated using the “vegan” package in R. Finally, Linear Discriminant Analysis Effect Size (LEfSe) was utilized to identify bacterial taxa exhibiting statistically significant differential abundance between groups, applying a threshold of an LDA score > 2.5 and *p* < 0.05.

### 2.6. Fecal Microbiota Transplantation Administration Experiment

Fresh fecal pellets (100 mg) were collected from donor mice in the CGA-treated group and suspended in 1 mL of sterile phosphate-buffered saline (PBS). The suspension was thoroughly homogenized by vigorous vortexing, and then passed through a sterile cell strainer (40 μm) to remove large particulate debris. Glycerol was added to the filtrate at a final concentration of 10% as a cryoprotectant. After thorough mixing, the suspension was aliquoted into sterile cryotubes and snap-frozen at −80 °C until use. Prior to transplantation, the frozen suspension was thawed and centrifuged at 10,000 rpm for 5 min at 4 °C. The supernatant (containing glycerol) was discarded, and the microbial pellet was resuspended in sterile PBS for immediate gavage. The FMT protocol was initiated by depleting the endogenous gut microbiota of the recipient mice. Eight-week-old female ICR mice were selected to ensure reproductive maturity and age consistency with the experimental cohorts. Following successful mating with male mice, the recipients were administered an antibiotic cocktail (ABX) in their drinking water for E5.5 days to achieve microbial reduction. The ABX solution consisted of 1 g/L streptomycin, 0.5 g/L ampicillin, 0.5 g/L vancomycin, and 1 g/L gentamicin, with the solution being refreshed daily. Recipient mice were assigned to three experimental groups (n = 6 per group): (1) the control group (ABX-CON), fed a standard diet and administered PBS; (2) the HS group (ABX-HS), subjected to the HS protocol and administered PBS; and (3) the HS + CGA FMT group (ABX-FMT), subjected to the HS protocol and orally gavaged daily with 0.2 mL of fecal microbiota suspension derived from CGA-treated donor mice. Antibiotic treatment was administered from E0.5 to E5.5 (6 days), and FMT gavage was performed from E6.5 to E11.5 (6 days). At E12.5, mice were euthanized, and intestinal, placental, and fetal tissues were harvested for subsequent downstream analysis.

### 2.7. Western Blot

Immunoblotting was performed according to standard protocols. Briefly, intestinal and placental tissues were lysed in RIPA buffer (Beyotime, Beijing, China) supplemented with 1% protease and phosphatase inhibitor cocktails. Protein concentrations were determined using a BCA Protein Assay Kit (Beyotime). Equivalent amounts of protein were resolved by SDS-PAGE and electrotransferred onto polyvinylidene fluoride (PVDF) membranes. After blocking with 5% non-fat milk, membranes were incubated with primary antibodies overnight at 4 °C. Protein bands were visualized using enhanced chemiluminescence (ECL) detection reagents and quantified by densitometric analysis using ImageJ software (version 1.54g, National Institutes of Health, Bethesda, MD, USA), with β-actin serving as the internal loading control. Primary antibodies used for Western blot analysis were as follows: rabbit anti-ZO-1 (1:2000—Proteintech, Wuhan, China, 21773-1-AP); rabbit anti-Occludin (1:2000—Proteintech, 27260-1-AP); rabbit anti-Claudin-1 (1:1000—Abmart, Beijing, China, TA0127); rabbit anti-Nrf2 (1:1000—Abcam, Cambridge, MA, USA, ab137550); mouse anti-Keap1 (1:1000—Abcam, ab226997); rabbit anti-HO-1 (1:1000—Abmart, T55113F); and mouse anti-β-actin (1:20,000—Abcam, ab8227, directly conjugated; no secondary antibody required).

### 2.8. Biochemical Assays

Tissue samples were homogenized in ice-cold PBS and centrifuged at 3000× *g* for 30 min at 4 °C. The resulting supernatants were collected for subsequent analysis. The levels of total antioxidant capacity (T-AOC) and malondialdehyde (MDA) were determined using commercial assay kits purchased from Beyotime Biotechnology (Shanghai, China). All analyses were performed strictly in accordance with the provided protocols.

### 2.9. Statistical Analysis

Statistical analyses were performed using SPSS (version 27.0, IBM Corp., Armonk, NY, USA), and graphical representations were generated with GraphPad Prism (version 10.0, GraphPad Software, San Diego, CA, USA). All independent biological replicates had a sample size of 6 mice per group. Data are presented as mean ± SEM for normally distributed data, or as median with 95% CI for non-normally distributed data. Group comparisons were performed using one-way ANOVA followed by Tukey’s post hoc test. To account for maternal effects, the average fetal/placental weight per dam was used as the experimental unit for analysis. A *p*-value < 0.05 was considered statistically significant. A *p*-value between 0.05 and 0.1 was considered to indicate a potential numerical trend, without reaching formal statistical significance.

## 3. Results

### 3.1. Chlorogenic Acid Rescues Heat Stress-Induced Fetal Growth Restriction

**Figure 1:**
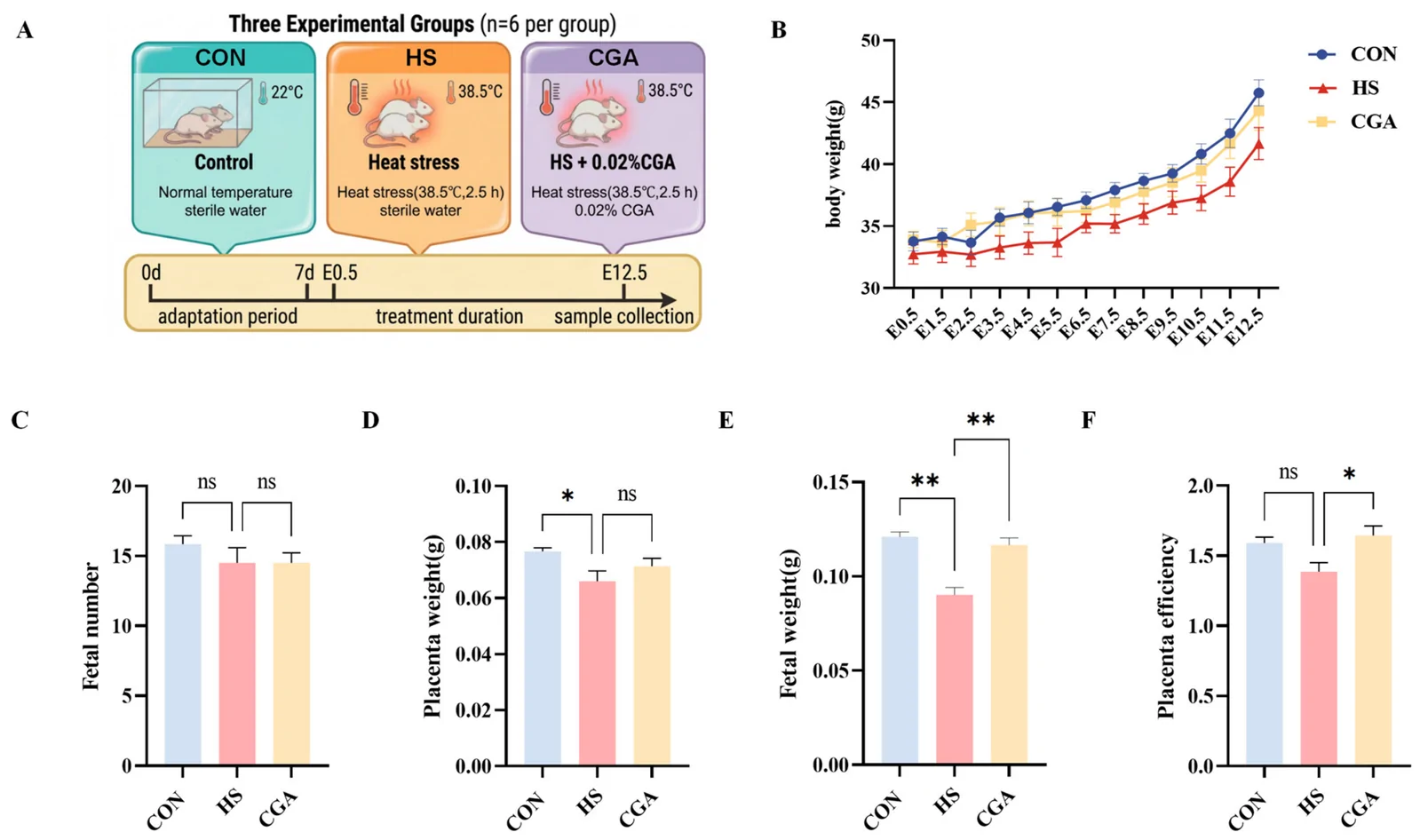
Chlorogenic acid alleviates heat stress-induced fetal growth restriction. (**A**) Schematic illustration of experimental design. (**B**) Body weight of pregnant mice during gestation in the CON, HS, and CGA groups. (**C**) Number of fetuses per pregnant mouse of the CON, HS, and CGA groups. (**D**–**F**) Placental (**D**) and fetal (**E**) weights and placenta efficiency (**F**) in the CON, HS, and CGA groups at E12.5 d. The data represent the mean ± SEM. * *p* < 0.05; ** *p* < 0.01; ns, not significant. All assays were performed with *n* = 6 mice per group.

To elucidate the reproductive consequences of climate change-mediated heat exposure, we employed a murine model of HS and explored the therapeutic potential of CGA. Pregnant dams underwent a daily 2.5 h HS regimen during the meridian hours (Figure 1A). While the relationship between litter size and FGR is well-documented in normative pregnancies, the synergistic effects of environmental stressors and litter size on growth outcomes remain contentious. To decouple HS-induced FGR from potential variations in litter size, we evaluated the relationship between litter size and fetal weight. Notably, maternal body weight changes and litter size remained consistent across all experimental cohorts (*p* > 0.05) (Figure 1B,C), yet a significant reduction in fetal weight was observed in the HS group relative to the CON and CGA groups (*p* < 0.05) (Figure 1D,E). Intriguingly, CGA treatment did not significantly alter placental weight compared to the HS group (*p* > 0.05). However, the HS-induced decline in the fetal-to-placental weight ratio—a definitive proxy for placental efficiency—was robustly reversed by CGA intervention (*p* < 0.05) (Figure 1F). Collectively, these data suggest that HS-driven FGR is primarily mediated by compromised placental efficiency, and that CGA supplementation serves as a potent strategy to mitigate these adverse pregnancy outcomes.

### 3.2. Chlorogenic Acid Rescues Heat Stress-Induced Fetal Growth Restriction by Restoring Placental Efficiency and Barrier Integrity

**Figure 2:**
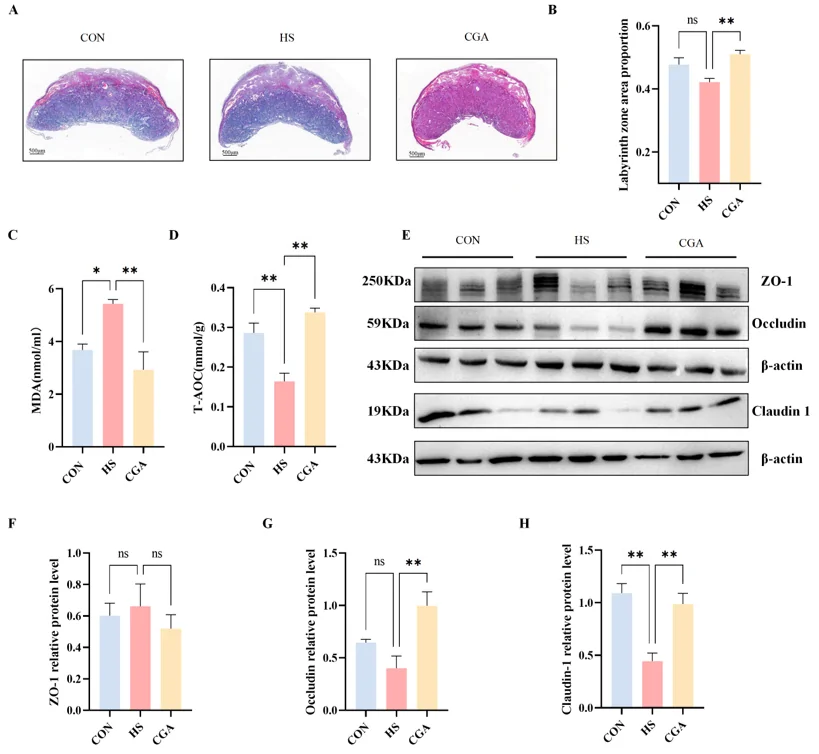
Chlorogenic acid alleviates placental oxidative stress and barrier disruption induced by heat stress. (**A**) Representative images of H&E-stained placental tissue sections. (**B**) The areal proportion of the labyrinth zone in the placenta. (**C**) The levels of MDA in the placenta tissues from different treated groups. (**D**) The levels of T-AOC in the placenta tissues from different treated groups. (**E**–**H**) Relative protein levels of ZO-1, Occludin, and Claudin-1 in the placenta. The data represent the mean ± SEM. * *p* < 0.05; ** *p* < 0.01; ns, not significant. All assays were performed with *n* = 6 mice per group.

To further elucidate the protective mechanisms of CGA against HS-induced placental injury, we systematically evaluated alterations in placental morphology, oxidative status, and barrier function. Histological analysis revealed that the total placental areas were comparable among the three groups (Figure 2A). However, HS exposure reduced the proportional area of the labyrinth zone relative to the total placental area, indicating impaired nutrient exchange at the maternal–fetal interface (*p* = 0.0646). Notably, CGA supplementation significantly attenuated this structural impairment induced by HS (*p* < 0.01) (Figure 2B). Regarding oxidative stress, HS treatment precipitated a significant decline in T-AOC alongside elevated MDA levels (*p* < 0.05) (Figure 2C,D), indicating exacerbated oxidative damage; however, CGA administration effectively reversed these trends and restored redox homeostasis (*p* < 0.01). Molecular investigations further revealed that HS significantly downregulated the critical tight junction protein Claudin-1 (*p* < 0.01), thereby compromising placental barrier integrity (Figure 2E–H). Conversely, treatment with CGA not only significantly rescued Claudin-1 expression (*p* < 0.01) but also synergistically upregulated Occludin (*p* < 0.01) (Figure 2G,H), reinforcing the tight junction framework across multiple dimensions. Collectively, our findings demonstrate that CGA effectively counters HS-induced placental injury through morphological restoration, redox balance, and molecular barrier reinforcement, which highlight its potential as a nutritional intervention strategy for mitigating HS during pregnancy.

### 3.3. CGA Mitigates Heat Stress-Induced Maternal Intestinal Barrier Impairment and Microbiota Dysbiosis

**Figure 3:**
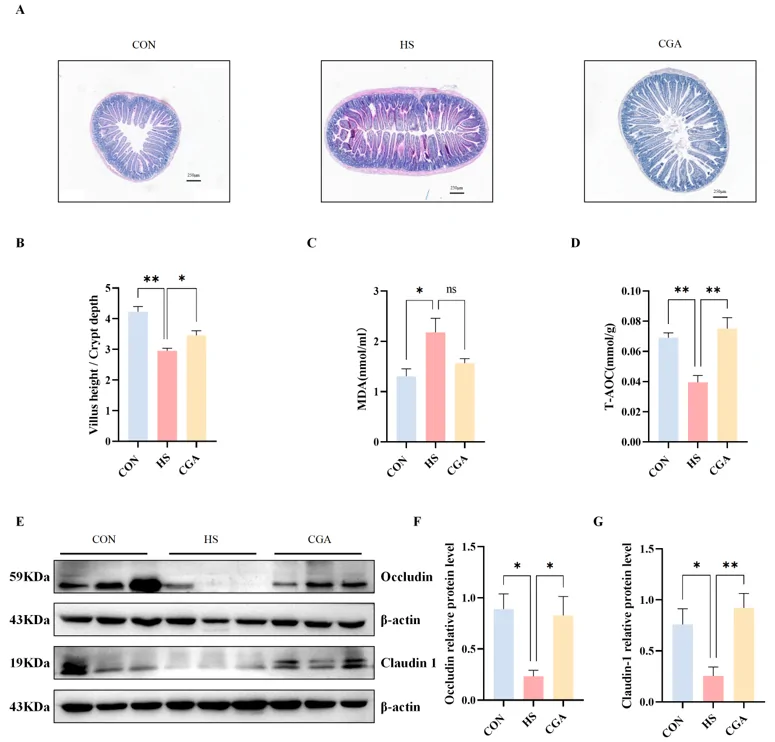
Gut oxidative stress and barrier disruption induced by heat stress can be alleviated by CGA. (**A**) Representative images of H&E-stained jejunum tissue sections. (**B**) The ratio of villous height to crypt depth of the jejunum tissue in the CON, HS, and CGA groups. (**C**) The levels of MDA in the colon tissues from different treated groups. (**D**) The levels of T-AOC in the colon tissues from different treated groups. (**E**–**G**) Relative protein levels of Occludin and Claudin-1 in the colon. The data represent the mean ± SEM. * *p* < 0.05; ** *p* < 0.01; ns, not significant. All assays were performed with *n* = 6 mice per group.

Consequently, we evaluated maternal intestinal integrity as a potential mediator of HS-induced damage. Histological examination of jejunal sections from pregnant mice revealed that the villus height-to-crypt depth ratio was significantly decreased in the HS group compared with the CON group (*p* < 0.01) (Figure 3A,B), indicating that HS exposure induced intestinal injury in the maternal jejunum. Notably, dietary CGA supplementation effectively attenuated this reduction (*p* < 0.05). Additionally, the MDA level in the maternal intestine of the HS group was significantly higher than that of the CON group (*p* < 0.05). In contrast, the T-AOC concentration was significantly decreased in the HS group compared with both the CON and CGA groups (*p* < 0.01), with MDA and T-AOC serving as definitive biomarkers of oxidative stress (Figure 3C,D). To further characterize barrier functionality, we quantified the abundance of key tight junction (TJ) proteins, including Occludin and Claudin-1. HS treatment drastically downregulated the expression of these proteins; however, these inhibitory effects were robustly reversed by CGA administration (*p* < 0.05) (Figure 3E–G). Collectively, these findings demonstrate that HS severely impairs maternal intestinal barrier function, an effect that is effectively mitigated by CGA treatment.

**Figure 4:**
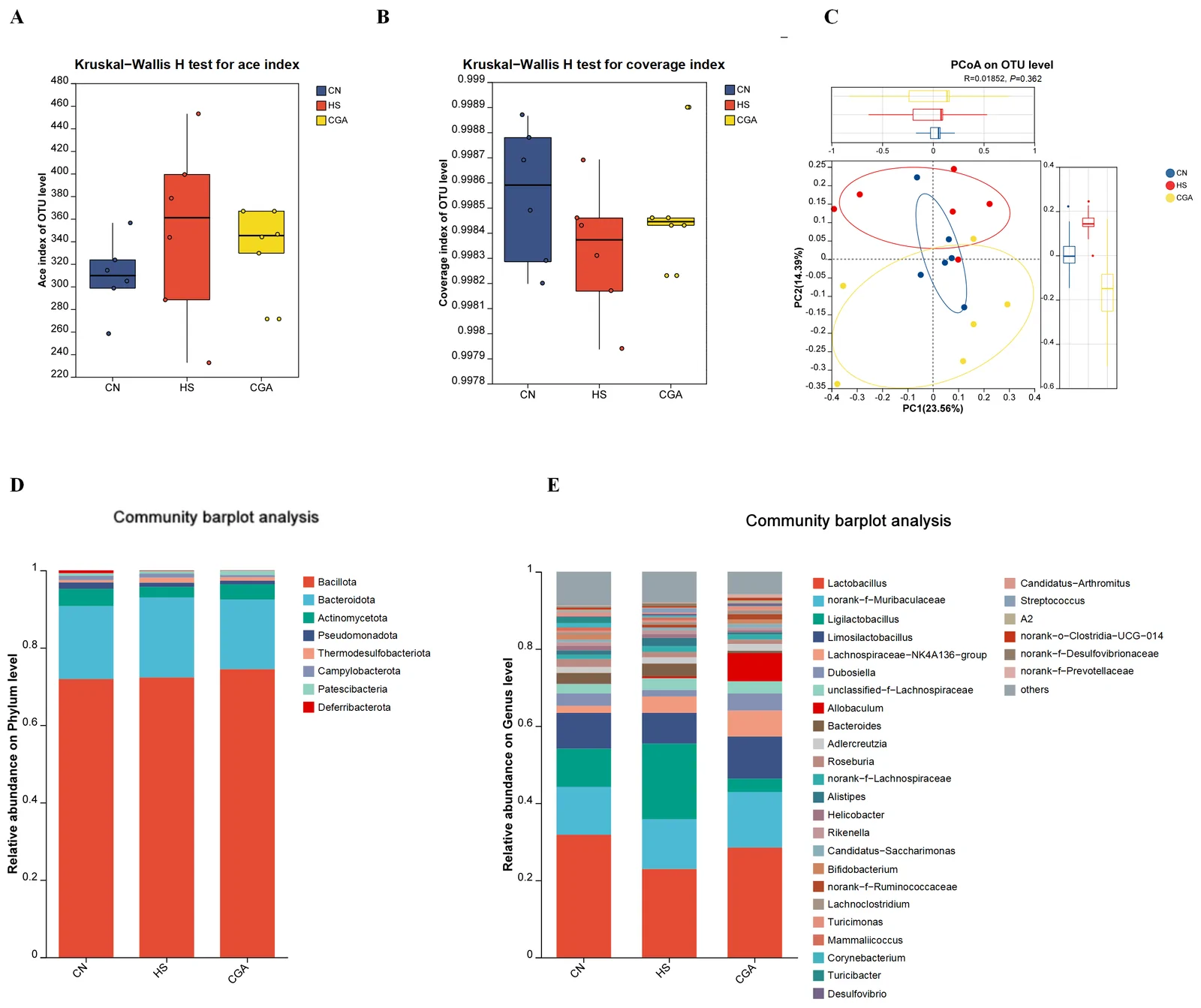
Intestinal microbiota in the CON, HS, and CGA mice. (**A**,**B**) The alpha-diversity indexes of ASVs among the CON, HS, and CGA groups. (**C**) The beta-diversity of ASVs among the CON, HS, and CGA groups. (**D**,**E**) Community barplot analysis showing the relative abundance of microbiota at different taxonomic levels. All assays were performed with *n* = 6 mice per group.

To determine whether the protective efficacy of CGA is mediated via gut microbiota modulation, we characterized the fecal microbial landscape using 16S rRNA high-throughput sequencing. α-diversity assessments, including Ace and Coverage indices, exhibited no significant deviations among the two cohorts, suggesting that global microbial richness and evenness remained stable (Figure 4A,B). Furthermore, principal Coordinate Analysis (PCoA) based on Bray–Curtis dissimilarity revealed no discrete clustering of global microbiota (*R* = 0.01852, *p* = 0.362), although CGA-treated samples displayed a subtle divergent trend along the PC2 axis (Figure 4C). At the phylum level, the intestinal landscape was predominantly composed of Firmicutes (~72–78%) and Bacteroidetes (~15–20%). Notably, CGA intervention normalized the HS-induced imbalance in the Firmicutes/Bacteroidetes (F/B) ratio, highlighting its capacity to fine-tune community structure without perturbing its primary composition (Figure 4D). Genus-level taxonomic analysis revealed that while the core microbiome, including *Lactobacillus*, *Ligilactobacillus*, and norank_f_Muribaculaceae, remained present, their relative abundances were differentially modulated (Figure 4E). Specifically, the suppression of *Lactobacillus* observed in the HS group (~23% vs. ~31% in the CON group) was not only reversed but significantly enhanced by CGA intervention (~37%), underscoring its potential as a prebiotic-like agent for beneficial taxa. Additionally, the CGA group exhibited a characteristic enrichment of *Limonisilactobacillus*, whereas *Ligilactobacillus* predominated in the HS group. In summary, our results support a model in which CGA maintains gut homeostasis not through broad-scale microbiota remodeling, but via the preferential expansion of specific beneficial Lactobacillus lineages.

### 3.4. Gut Microbiota Mediates CGA-Induced Improvements in Placental Function and Systemic Barrier Integrity

**Figure 5:**
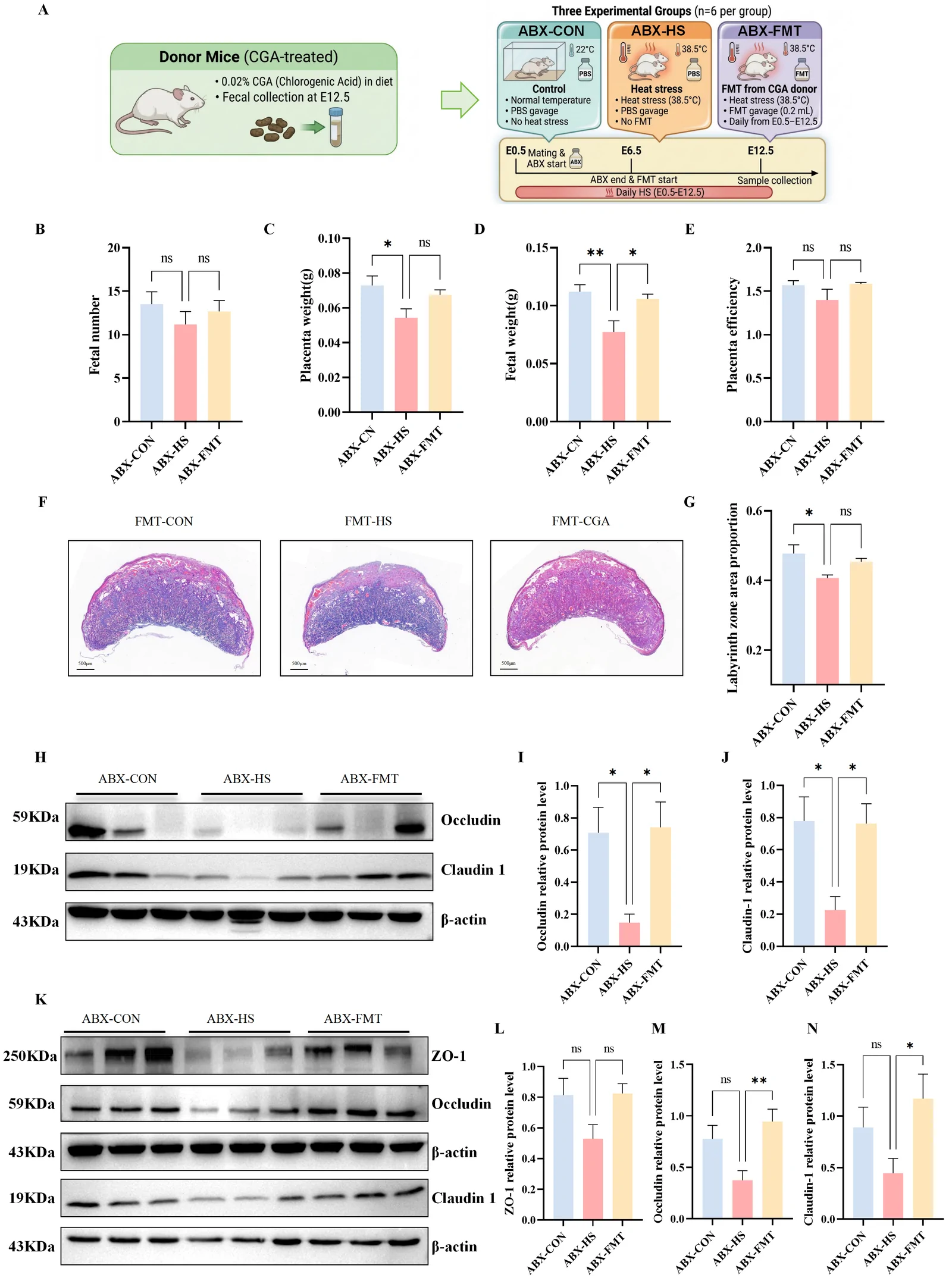
Pseudo-sterile experiment verifies the protective effect of CGA on HS-induced reproduction impairment through regulating gut microbiota. (**A**) Schematic illustration of experimental design. (**B**) Number of fetuses per pregnant mouse of the ABX-CON, ABX-HS, and ABX-FMT groups. (**C**–**E**) Placental and fetal weights and placenta efficiency in the ABX-CON, ABX-HS, and ABX-FMT groups at 12.5 days of gestation. (**F**) Representative images of H&E-stained placental tissue sections. (**G**) The areal proportion of the labyrinth zone in the placenta. (**H**–**J**) Relative protein levels of Occludin and Claudin-1 in the colon. (**K**–**N**) Relative protein levels of ZO-1, Occludin, and Claudin-1 in the placenta. The data represent the mean ± SEM. * *p* < 0.05; ** *p* < 0.01; ns, not significant. All assays were performed with *n* = 6 mice per group.

To determine whether the gut microbiota is essential for the CGA-mediated rescue of HS-induced reproductive damage, we employed an antibiotic-induced ABX mouse model. Following a 6-day antibiotic reduction phase, the mice were divided into three cohorts: ABX-CON, ABX-HS, and ABX-FMT. The ABX-CON and ABX-HS groups were administered oral PBS, whereas the ABX-FMT group underwent microbial reconstitution via FMT from CGA-supplemented donors (Figure 5A). Our data demonstrate that with the same number of live fetuses, the antibiotic-treated group (ABX-HS) led to a significant reduction in both fetal and placental weights compared to the antibiotic control group (ABX-CON). Notably, FMT from CGA-treated donors effectively alleviated these adverse effects and significantly restored fetal weight (*p* < 0.05) (Figure 5D). Concurrently, we performed H&E staining of placental tissues and quantified the area proportion of the labyrinth zone (Figure 5F,G). Statistical analysis revealed that the labyrinth zone area proportion was significantly reduced in the ABX-HS group compared with the ABX-CON group (*p* < 0.05), while a trend toward restoration was observed in the ABX-FMT group (*p* = 0.1435), indicating improved placental functional capacity. These findings align with our earlier phenotypic observations and reinforce the critical role of the gut microbiota in maintaining developmental resilience. This suggests that the microbiota–CGA axis alleviates fetal growth restriction primarily by optimizing nutrient transfer at the maternal–fetal interface, rather than by promoting structural expansion of the placenta.

Further molecular investigations into the gut–placenta axis revealed that HS-induced microbial dysbiosis is closely associated with impaired barrier integrity. Specifically, the expression of intestinal tight junction proteins (Claudin-1 and Occludin) was significantly downregulated in the ABX-HS group (*p* < 0.05) (Figure 5H–J). Concurrently, gestational HS exacerbated oxidative stress and compromised placental barrier function, as indicated by the downregulation of placental tight junction proteins (Figure 5K–N). Crucially, FMT intervention successfully reversed these molecular defects (*p* < 0.05). In summary, these data suggest that the microbiota regulated by CGA may protect reproductive health by enhancing the integrity of system barriers and inhibiting oxidative stress.

### 3.5. CGA Activates the Keap1-Nrf2 Antioxidant Signaling Pathway in a Microbiota-Dependent Manner

**Figure 6:**
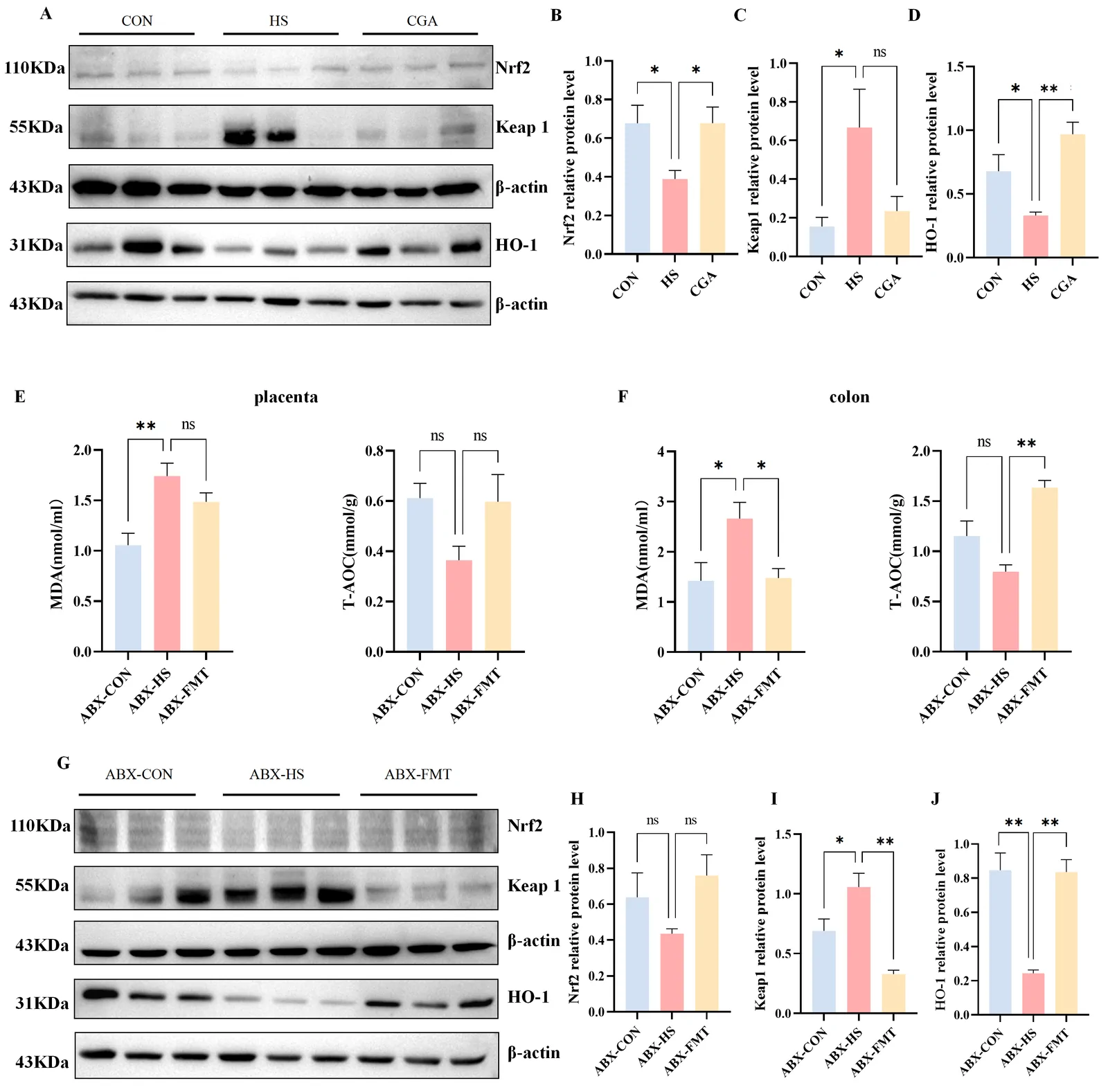
CGA and CGA-derived gut microbiota alleviate oxidative stress by activating the Nrf2-Keap1 pathway. (**A**–**D**) Relative protein levels of Nrf2, Keap1, and HO-1 in the placenta. (**E**) The levels of MDA and T-AOC in the placenta tissues from different treated groups. (**F**) The levels of MDA and T-AOC in the colon tissues from different treated groups. (**G**–**J**) Relative protein levels of Nrf2, Keap1, and HO-1 in the placenta. The data represent the mean ± SEM. * *p* < 0.05; ** *p* < 0.01; ns, not significant. All assays were performed with *n* = 6 mice per group.

Building upon our identification of the microbiota–CGA reproductive protection axis, we sought to elucidate its downstream molecular mechanisms. Given that oxidative stress is a central pathological driver of HS-induced placental damage, we focused on the Keap1-Nrf2 antioxidant signaling pathway, the primary regulatory hub for cellular responses to oxidative stress. As illustrated in Figure 6A–D, HS exposure significantly upregulated the expression of the negative regulator Keap1 (*p* < 0.05), while concomitantly downregulating the protein levels of the key transcription factor Nrf2 and its downstream target protein HO-1 (*p* < 0.05), indicating profound suppression of the antioxidant defense system. Notably, dietary intervention with CGA effectively reversed these alterations: Keap1 expression was significantly suppressed, returning to baseline levels (*p* = 0.068), whereas Nrf2 and HO-1 expression were significantly upregulated (*p* < 0.05). These findings suggest that CGA activates the cellular antioxidant response by preventing Keap1-mediated Nrf2 degradation, thereby mitigating HS-induced oxidative damage. This molecular mechanism aligns with the improvements in placental oxidative stress markers (T-AOC and MDA) observed in our earlier analyses.

To determine whether the regulation of the Keap1-Nrf2 pathway depends on microorganisms, we used the ABX model to evaluate the molecular markers of this pathway in parallel. These results demonstrated that the ABX-HS group exhibited hallmarks of oxidative stress in both the placenta and the intestine, and the ABX-FMT group showed certain antioxidant capacity (Figure 6E,F). Crucially, the ABX-FMT group, which received FMT from CGA-treated donors, recapitulated the molecular phenotype observed with direct CGA intervention. In this cohort, Keap1 expression was significantly suppressed and HO-1 levels were substantially restored, achieving a magnitude of effect comparable to that of the direct CGA treatment group (*p* < 0.05) (Figure 6G–J). These findings indicate that microbial transplantation alone is sufficient to recapitulate the antioxidant-activating effects of CGA, and further suggest that the regulatory effect of CGA on the Keap1-Nrf2 pathway may be mediated by the gut microbiota.

## 4. Discussion

Amplified by global climate change, HS now constitutes a pervasive environmental hazard that severely disrupts mammalian reproductive success and perturbs fetal developmental trajectories [28]. While the detrimental effects of gestational HS on FGR are well-documented, the underlying mechanisms and viable therapeutic interventions remain insufficiently understood. In the present study, we elucidate a novel protective paradigm: CGA effectively ameliorates HS-induced FGR and placental dysfunction. Critically, we demonstrate that this protective effect is not solely a direct systemic action of CGA, but is mediated, at least in part, by the maternal gut microbiota and the subsequent activation of the host Keap1-Nrf2 antioxidant defense system.

A hallmark of gestational HS in our model was the simultaneous impairment of both the maternal intestinal and placental barriers. HS significantly decreased the expression of key tight junction proteins (Claudin-1 and Occludin) in the intestine and placenta, leading to intestinal barrier impairment accompanied by severe oxidative stress. The concept of the “gut–placenta axis” posits that compromised maternal intestinal integrity allows endotoxins and pro-inflammatory mediators to translocate into systemic circulation, ultimately arriving at the maternal–fetal interface to induce placental damage [29,30]. Previous studies have demonstrated that CGA exerts beneficial effects on intestinal barrier integrity [31]. In the present study, we further show that under HS conditions, CGA effectively reinforces both the intestinal and placental barriers. These findings suggest that maintaining maternal intestinal integrity is a prerequisite for protecting the placenta from systemic oxidative stress and inflammatory cascades.

A pivotal discovery of our study is the causal role of the gut microbiota in executing the protective effects of CGA. While global microbial diversity remained relatively stable, CGA exerted a targeted modulatory effect, normalizing the Firmicutes/Bacteroidetes ratio and specifically enriching beneficial Lactobacillus lineages. Members of the Lactobacillus genus are renowned for their capacity to produce short-chain fatty acids (SCFAs) and lactic acid, which are critical for maintaining intestinal luminal pH, nourishing colonocytes, and reinforcing tight junctions [32,33]. The essentiality of this microbial shift was unequivocally proven by our antibiotic-induced ABX and FMT models. Mice that received microbiota from CGA-treated donors (ABX-FMT) successfully recapitulated the phenotypic and molecular rescue observed with direct CGA supplementation. They exhibited restored placental efficiency and enhanced systemic barrier function despite active HS exposure. This confirms that CGA functions largely as a prebiotic-like modulator, restructuring specific microbial niches to confer systemic resilience to the host.

Accumulating evidence establishes oxidative stress as the central pathological driver linking environmental heat exposure to cellular and tissue damage. Prolonged hyperthermia disrupts the delicate equilibrium between ROS production and scavenging, triggering a cascade of lipid peroxidation, protein denaturation, and ultimately, the breakdown of critical cellular barriers [34]. Our molecular investigations revealed that the Keap1-Nrf2 signaling pathway is the primary regulatory hub responding to this stress. HS caused a profound suppression of the antioxidant defense system, evidenced by the upregulation of the negative regulator Keap1 and the subsequent downregulation of Nrf2. Remarkably, CGA treatment reversed this suppression, an effect that was brilliantly mirrored in the ABX-FMT cohort. The regulatory role of the gut microbiota in the Keap1-Nrf2 pathway serves as a mechanistic bridge between the intestinal lumen and systemic maternal–fetal health [35,36,37]. We postulate that specific microbiota-derived metabolites, such as SCFAs acting as histone deacetylase (HDAC) inhibitors, or CGA-derived phenolic electrophiles, act as systemic signaling molecules. Upon traversing the fortified intestinal barrier, these metabolites chemically modify critical cysteine residues on Keap1 (e.g., Cys151), inducing a conformational change that prevents Nrf2 degradation. Once stabilized, Nrf2 translocates into the nucleus to drive the transcription of endogenous antioxidant enzymes, thereby neutralizing HS-induced ROS at the maternal–fetal interface and rescuing placental nutrient exchange capabilities.

Together, these findings position the maternal gut microbiome as a highly modifiable target for protecting fetal development against climate-driven thermal stress. Although CGA represents a highly promising and safe nutritional intervention, whether it exerts similar protective effects under normal physiological conditions remains to be elucidated, and future studies incorporating a CON + CGA control group are warranted to address this question. Furthermore, the precise mechanisms by which CGA modulates the Keap1-Nrf2 signaling pathway require identification of the key microbial metabolites responsible for pathway activation through untargeted metabolomics. Concurrently, the molecular mechanisms underlying CGA-mediated regulation of this pathway warrant further validation at the cellular level. Verification of these mechanisms in germ-free (GF) mouse models and subsequent human cohort studies will be critical steps toward translating microbiota-targeted nutritional strategies into clinical obstetric care.

## 5. Conclusions

Collectively, our study maps a comprehensive pathway from environmental stressors to molecular mechanisms. We propose that gestational HS-induced microbial dysbiosis and intestinal barrier impairment may contribute, at least in part, to systemic oxidative stress and placental insufficiency. Dietary supplementation with CGA effectively intercepts this pathological cascade by remodeling the gut microbiota, which in turn fortifies the gut–placental barrier axis and mediates the Keap1-Nrf2 antioxidant pathway. These findings not only advance our understanding of microbiota–host crosstalk during complicated pregnancies but also highlight CGA as a highly promising, accessible nutritional intervention to safeguard reproductive health in an era of global warming.

## Data Availability

The 16S rRNA sequencing data generated in this study have been deposited in the NCBI Sequence Read Archive (SRA) under accession number PRJNA1477418. These data will be made publicly available upon publication. All other data supporting the reported results are contained within the article and its Supplementary Materials.

## Supplementary Materials

The following supporting information can be downloaded at: https://www.mdpi.com/article/10.3390/antiox15081036/s1, Table S1. Colonic T-AOC data from the CGA trial; Table S2. Jejunal villus morphometric statistics from the CGA trial; Table S3. MDA data from the CGA trial; Table S4. Placental T-AOC data from the CGA trial; Table S5. Litter data from the CGA trial; Table S6. Placental area morphometric statistics; Table S7. MDA data from the FMT-CGA trial; Table S8. T-AOC data from the FMT-CGA trial; Table S9. Litter data from the FMT-CGA trial.

## Abbreviations

ABX: antibiotic cocktail; ASV: amplicon sequence variant; CGA: chlorogenic acid; GF: germ-free; ECL: chemiluminescence; F/B: Firmicutes/Bacteroidetes; FGR: fetal growth restriction; FMT: fecal microbiota transplantation; HDAC: histone deacetylase; H&E: hematoxylin and eosin; HS: heat stress; LEfSe: Linear discriminant analysis Effect Size; LPS: lipopolysaccharides; LZ: labyrinth zone; MDA: malondialdehyde; PCoA: Principal Coordinate Analysis; PVDF: polyvinylidene fluoride; ROS: reactive oxygen species; rRNA: Ribosomal RNA; SCFAs: short-chain fatty acids; sIgA: secretory immunoglobulin A; T-AOC: total antioxidant capacity; TJ: tight junction; ZO-1: Zonula Occludens-1.
